# PML-RAR alpha induces the downmodulation of HHEX: a key event responsible for the induction of an angiogenetic response

**DOI:** 10.1186/s13045-016-0262-5

**Published:** 2016-04-07

**Authors:** Ernestina Saulle, Alessia Petronelli, Elvira Pelosi, Elena Coppotelli, Luca Pasquini, Ramona Ilari, Francesco Lo-Coco, Ugo Testa

**Affiliations:** Department of Hematology, Oncology and Molecular Medicine, Istituto Superiore di Sanità, Rome, Italy; Department of Biomedicine and Prevention, University of Rome “Tor Vergata” and Fondazione Santa Lucia, Rome, Italy

## Abstract

**Background:**

Recent studies indicate that angiogenesis is important in the pathogenesis of acute myeloid leukemias (AMLs). Among the various AMLs, the bone marrow angiogenetic response is particularly pronounced in acute promyelocytic leukemia (APL). However, the molecular mechanisms responsible for this angiogenetic response are largely unknown. In the present study, we have explored the role of HHEX, a homeodomain transcription factor, as a possible mediator of the pro-angiogenetic response observed in APL. This transcription factor seems to represent an ideal candidate for this biologic function because it is targeted by PML-RARα, is capable of interaction with PML and PML-RARα, and acts as a regulator of the angiogenetic response.

**Methods:**

We used various cellular systems of APL, including primary APL cells and leukemic cells engineered to express PML-RARα, to explore the role of the PML-RARα fusion protein on HHEX expression. Molecular and biochemical techniques have been used to investigate the mechanisms through which PML-RARα downmodulates HHEX and the functional consequences of this downmodulation at the level of the expression of various angiogenetic genes, cell proliferation and differentiation.

**Results:**

Our results show that HHEX expression is clearly downmodulated in APL and that this effect is directly mediated by a repressive targeting of the HHEX gene promoter by PML-RARα. Studies carried out in primary APL cells and in a cell line model of APL with inducible PML-RARα expression directly support the view that this fusion protein through HHEX downmodulation stimulates the expression of various genes involved in angiogenesis and inhibits cell differentiation.

**Conclusions:**

Our data suggest that HHEX downmodulation by PML-RARα is a key event during APL pathogenesis.

**Electronic supplementary material:**

The online version of this article (doi:10.1186/s13045-016-0262-5) contains supplementary material, which is available to authorized users.

## Background

The hematopoietic expressed homeobox gene (HHEX), also known as proline-rich homeodomain (PRH), is a transcription factor containing the DNA-binding domain termed the homeodomain. Similarly to the homeobox proteins, HHEX regulates cell development and differentiation, being required for the formation of the vertebrate body axis and the hematopoietic and vascular systems [1]. HHEX^−/−^ mice display embryonic lethality due to impaired forebrain, liver, and thyroid development; these mice display also defective vasculogenesis and elevated VEGF-A levels [2, 3].

HHEX is expressed in areas of the mammalian embryos that mainly contribute to hematopoietic and vascular development [1]. In particular, HHEX expression is seen very early during embryonic development in the blood islands of the yolk sac [4]. HHEX is highly expressed in stem cells and myeloid and lymphoid progenitors and its expression is maintained in adult hematopoietic tissues at the level of several blood cell lineages, including hematopoietic progenitors, lymphocytes, and myeloid lineages [5, 6]. Importantly, HHEX expression was found to be downregulated during terminal differentiation of both B cells [1] and myeloid cells [7]. In fact, using Myb-Ets-transformed chicken blastoderm cells (MEPs), it was shown that HHEX RNA and protein levels are downregulated when MEPs differentiate along the myelomonocytic and erythrocytic lineages, while they are maintained when these cells differentiate toward the thrombocytic lineage [7]. Furthermore, HHEX expression is downmodulated also in the T-cell lineage and this downregulation is physiologically critical since HHEX overexpression in these cells determines the development of T-cell leukemia in mice [8]. Using various embryonic stem cell differentiation models, it was possible to show that HHEX is required for proliferation and differentiation of definitive HSCs [9–11]. Particularly, Paz and coworkers have shown that HHEX^−/−^ embryonic stem cells when triggered to hematopoietic differentiation display the accumulation of early hematopoietic progenitors CD41^+^c-kit^+^ and a reduced capability to generate myeloid hematopoietic colonies, such as BFU-Mix, BFU-E, and CFU-GM [11].

Few studies have explored the expression and a possible deregulation of HHEX in leukemic cells. HHEX was expressed in the large majority of leukemic cell lines and its expression is usually lost when these cell lines are induced to differentiate [12]. In some rare AML patients, it was reported that a specific double translocation involving nucleoporin 98 was fused to the DNA-binding domain of the HHEX transcription factor [13]. The mechanism resulting in leukemia in these patients is not known, but it was proposed that the fusion protein may compete with endogenous HHEX for HHEX targets and may derepress genes normally blocked by HHEX [13].

Importantly, HHEX was shown to interact with the promyelocytic leukemia protein (PML) in various leukemic cell lines, including the promyelocytic cell line NB4 [14]. Yeast two-hybrid experiments have shown that HHEX was capable of directly interacting with PML across its ring finger domain, which is required for the protein activity in the control of cell growth [14]. Furthermore, HHEX was shown to be able to interact also with the PML-RARα oncoprotein that characterizes acute promyelocytic leukemias (APLs) [14]. According to these observations, it was proposed that disruption not only of PML but also of HHEX functions by PML-RARα fusion protein may play a relevant role in the pathogenesis of APLs [14]. In an attempt to define the mechanism through which PML-RARα blocks myeloid differentiation at the promyelocytic stage, Wang and coworkers have shown that PML-RARα targets promoter regions containing PU.1 consensus and RARE half sites in APL cells [15]. Among the various gene promoters displaying these characteristics, there is also the HHEX promoter, seemingly repressed by PML/RARα binding [15]. PML-RARα-mediated repression of PU.1-mediated transactivation was restored by the addition of all-trans retinoic acid (ATRA). The key functional role of PML-RARα-mediated repression of PU.1 expression and function was carefully confirmed by the same authors in other studies [16, 17].

Given the key role of HHEX in the control of hematopoietic cell differentiation, the targeting of the HHEX gene by the fusion oncoprotein PML-RARα, and the capacity of the HHEX protein to interact with PML and PML-RARα, we sought to investigate the expression and the possible deregulation of HHEX in APLs. In the present study, we have explored the expression and the deregulation of HHEX in APL. Our results indicate that HHEX expression is clearly downmodulated in APLs, while VEGF-A expression is upregulated. The study of an APL cell line model with inducible PML-RARα expression supports the view that this fusion protein significantly downmodulates HHEX expression. The inhibitory effect exerted by PML-RARα on HHEX expression seems to be physio-pathologically relevant to mediate the inhibitory effect on cell differentiation and the pro-angiogenetic effect induced by this fusion protein.

## Results

### HHEX expression is downmodulated during granulocytic differentiation

In a previous study, Wang and coworkers have shown that PML/RARα acts as a potent repressor of PU.1-RARE binding sites present at the level of various genes, including HHEX gene; this repression was relieved by all-trans retinoic acid (ATRA) [15]. Given the key role of HHEX as a repressor of various angiogenetic genes and the elevated expression of angiogenetic factors in APL, it seemed of interest to explore in detail the possible consequences of a deregulated HHEX expression in APLs induced by PML-RARα.

**Fig. 1:**
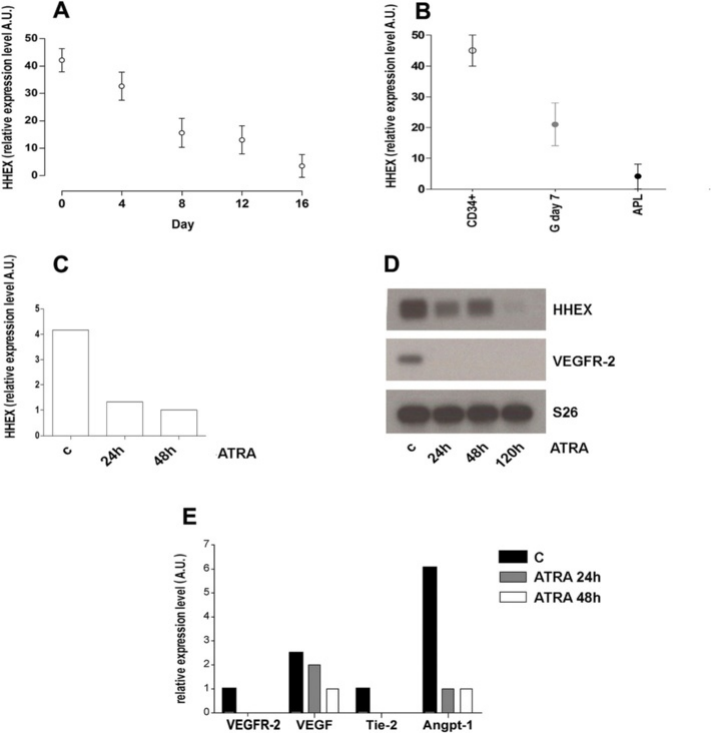
HHEX expression during granulocytic differentiation of normal CB CD34^+^ cells (**a**, **b**) and of primary APL cells (**b**, **c**); expression of angiopoietic growth receptors (VEGF-R2 and Tie-2) and ligands (VEGF and angiopoietin-1) in APL primary cells induced to granulocytic differentiation (**d**, **e**). Normal CD34^+^ cells have been purified from cord blood and grown in vitro under cell culture conditions allowing their selective granulocytic differentiation; at different days of culture, cell aliquots were harvested and processed for HHEX expression by real-time PCR analysis. Primary leukemic APL blasts were purified from the bone marrow of an APL patient and grown in vitro in cell culture medium containing 1 μM ATRA, harvested at different times and processed for HHEX, VEGF, VEGF-R2, Tie-2, and angiopoietin-1 expression by real-time PCR

Thus, in a first set of experiments, we evaluated HHEX messenger RNA (mRNA) expression by real-time PCR in human CD34^+^ cells triggered to selective granulocytic differentiation under appropriate and selective cell culture conditions. HHEX was clearly expressed in undifferentiated CD34^+^ progenitors and its expression progressively and continuously decreased during the process of granulocytic differentiation and maturation (Fig. 1a).

### HHEX expression is downmodulated in APLs

We then compared the level of HHEX mRNA expression observed in either normal CD34^+^ cells or day 7 granulocytic cells, composed by a majority of normal promyelocytes, with the HHEX mRNA levels observed in fresh diagnostic APL blasts and we observed that HHEX levels were markedly lower in leukemic cells than in their normal counterpart (Fig. 1b). In APL fresh leukemic blasts induced in vitro to terminal granulocytic differentiation, a further decrease of HHEX RNA expression was observed, its expression being virtually absent in terminally differentiated APL cells (i.e., at 120 h after ATRA addition) (Fig. 1c). ATRA also induced a marked downmodulation of the expression of various angiogenetic genes, including VEGF-R2 (Fig. 1d).

**Fig. 2:**
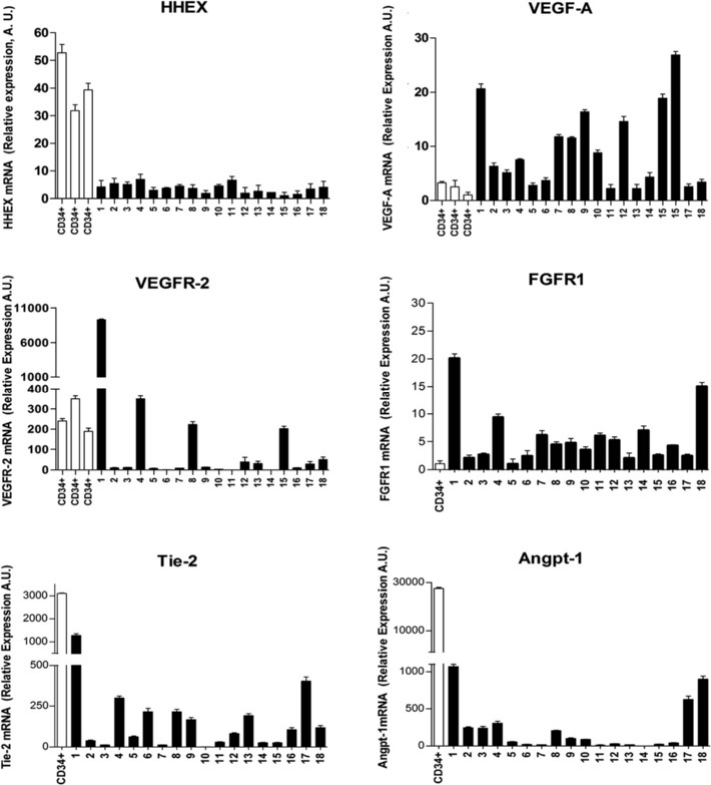
Analysis of HHEX, VEGF-A, VEGFR-2, FGFR1, Tie-2, and Angp-1 mRNA levels in normal CB CD34^+^ cells and in leukemic blasts of 18 primary APLs. The expression levels of the mRNA of these genes were evaluated by real-time PCR, and the results reported in the figure represent the mean ± SEM values observed for individual analyses repeated three times

In a second set of experiments, we evaluated the expression of HHEX and of several angiogenetic genes, including VEGF-A, VEGF-R2, angiopoietin-1, Tie-2, and FGFR-1, in APL fresh blasts derived from 18 patients. The majority of these angiogenetic genes and, particularly, VEGF-A, Tie-2, and FGFR-1 were frequently expressed in APL cells, while HHEX was scarcely expressed in all APL patients, compared to normal CD34^+^ cells (Fig. 2). However, it is worth noting that HHEX levels are heterogeneous in distinct APL patients and we hypothesized a possible negative correlation between HHEX and VEGF-A levels. The plotting of individual HHEX and VEGF-A levels showed the existence of a negative correlation between these two parameters (*p* < 0.04).

In order to determine whether low HHEX expression levels were restricted to specific FAB AML subtypes, the RNA seq data from TCGA samples were correlated with each FAB subtype. Within each FAB category, the samples were ordered according to the expression levels of respectively HHEX and VEGF-A transcripts (Additional file 1: Figure S1A). This analysis showed that (a) among the various AMLs, M3 (APLs), M4, and M5 are those expressing the lowest HHEX levels, compared to M0, M1, and M2 AMLs; (b) the levels of VEGF-A expression were much higher in M3 than in all the other FAB AML subtypes; (c) the HHEX/VEGF-A ratio was much lower in M3 than in the other FAB AML subtypes; (d) the plotting of the individual HHEX and VEGF-A levels in M3 AML patients showed the existence of a significant negative correlation between these two parameters (*p* < 0.028) (Additional file 1: Figure S1B). Since HHEX levels were particularly low in M5 AMLs, it was of interest to verify whether there is also among these AMLs and inverse relationship between HHEX and VEGF-A levels. The results of this analysis showed the existence of a significant negative correlation between HHEX and VEGF-A levels (Additional file 1: Figure S1C).

### PML-RARα downmodulates HHEX expression

**Fig. 3:**
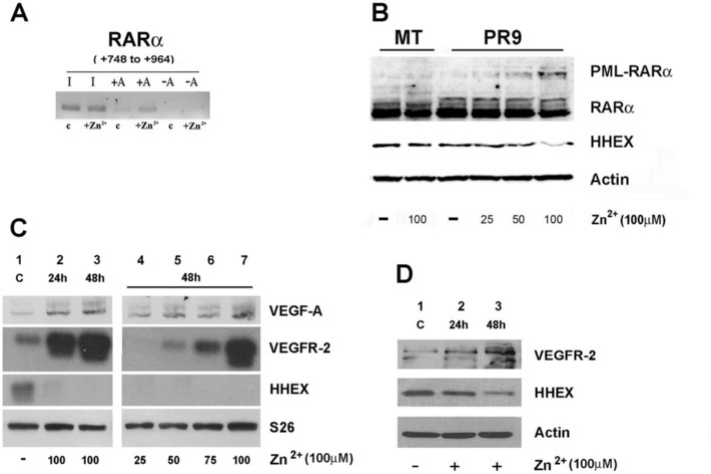
Effect of PML/RARα induction on the binding of PML-RARα to the HHEX gene promoter and on the expression of VEGF-A, VEGFR-2, and HHEX at RNA and at protein level. **a** PML-RARα binding to the HHEX promoter as shown by ChIP experiments. Nuclear extracts derived from PR9 cells grown for 24 h either in the absence (**c**) or in the presence of ZnSO_4_ (Zn^2+^); cells were crosslinked in vivo; after cell lysis, chromatin fragments were immunoprecipitated with anti-RARα antibody; and after DNA purification, DNA regions containing HHEX were amplified. In the figure, the HHEX PCR signal without immunoprecipitation is indicated as −A (IgG control) and, after immunoprecipitation, as +A (RARα antibody). **b**, **c**, **d** ZnSO_4_(Zn^2+^) was added to PR9 cells and, at different time points and concentrations, cell aliquots were harvested and processed for evaluation of PML-RARα expression at protein level (**b**) and of VEGF-A, VEGFR-2, and HHEX expression at RNA level by RT-PCR (**c**) and at protein level by Western blotting (**d**). S26 was used for RT-PCR and β-actin for Western blotting normalization

**Fig. 4:**
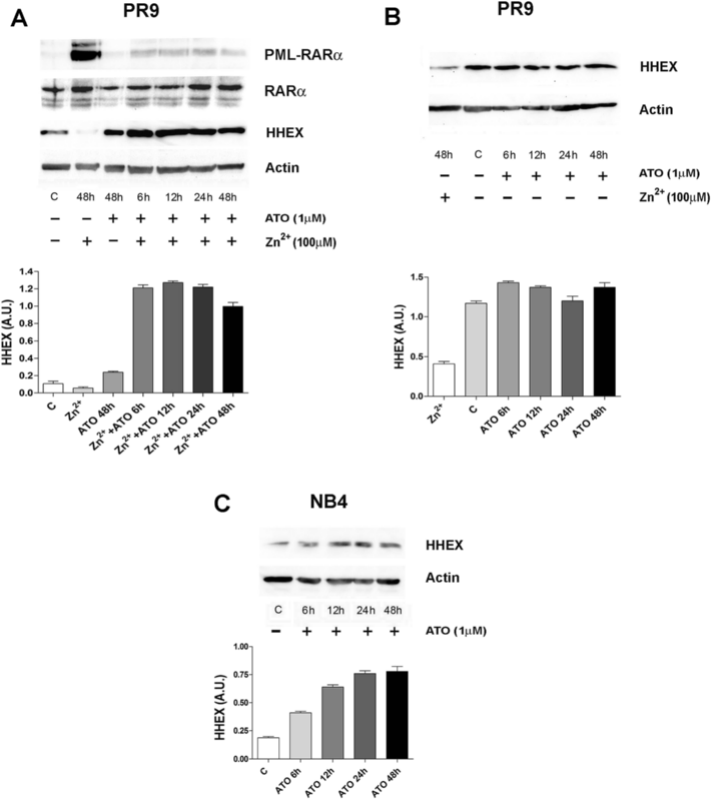
Effect of ATO on downmodulation of HHEX expression elicited by PML-RARα induction in PR9 cells (*top* and *middle panels*) and on HHEX expression observed in NB4 cells (*bottom panel*). *Top panels* (**a**, **b**): PR9 cells have been grown either in the absence (**c**) or in the presence of ZnSO_4_ for 48 h (*left and right panels*), in the presence of ATO for different times (from 6 to 48 h, *right panel*), or in the presence of ZnSO_4_ + ATO for different time points (from 6 to 48 h, *left panel*) and HHEX, PML-RARα, or RARα expression were assessed by WB. A representative WB is shown at the *top* of each panel and the quantitative evaluation (mean ± SEM in three separate experiments). *Bottom panel* (**c**): NB4 cells have been grown either in the absence (**c**) or in the presence of ATO for different times (from 6 to 48 h) and HHEX expression was analyzed by WB

In order to evaluate the molecular mechanisms through which PML-RARα induces a downmodulation of HHEX expression and the possible functional consequences of this downmodulation, we have used as an experimental tool U-937 cells transfected with PML-RARα (clone PR9) [18]. In line with a previous report [15], CHIP experiments showed that PR9 cells treated with Zn^2+^ PML-RARα were able to bind at the level of a RARα binding site present in the HHEX gene regulatory region (first exon) (Fig. 3b). In a preliminary experiment, we have shown that the activation of PML-RARα expression in PR9 cells (in these cells, the PML-RARα gene is under the control of the metallothionein promoter; and thus, its expression is inducible by Zn^2+^ addition) induced by Zn^2+^ addition resulted in a marked decline of HHEX expression (Fig. 3b), paralleled by a concomitant induction of the expression of angiogenetic genes, such as VEGFR-2: at day 4 after Zn^2+^ addition, HHEX expression was virtually undetectable at mRNA level and very low at protein level, compared to the levels observed in control untreated PR9 cells (Fig. 3c, d). In order to provide more direct evidence that the downmodulation of HHEX observed in Zn^2+^-treated cells was really related to PML/RARα expression, we have treated PR9 cells with Zn^2+^ and arsenic trioxide (ATO), an agent promoting PML-RARα degradation (Fig. 4a). These experiments showed that ATO added together with Zn^2+^ completely prevented the Zn^2+^-induced HHEX downmodulation in PR9 cells (Fig. 4a, b). The causal link between PML-RARα expression and low HHEX expression was further reinforced by the observation that treatment of NB4 cells with ATO elicited a pronounced upmodulation of HHEX expression (Fig. 4c). Thus, PR9 cells seemed to be a suitable model to explore the modulation of HHEX expression by PML-RARα.

**Fig. 5:**
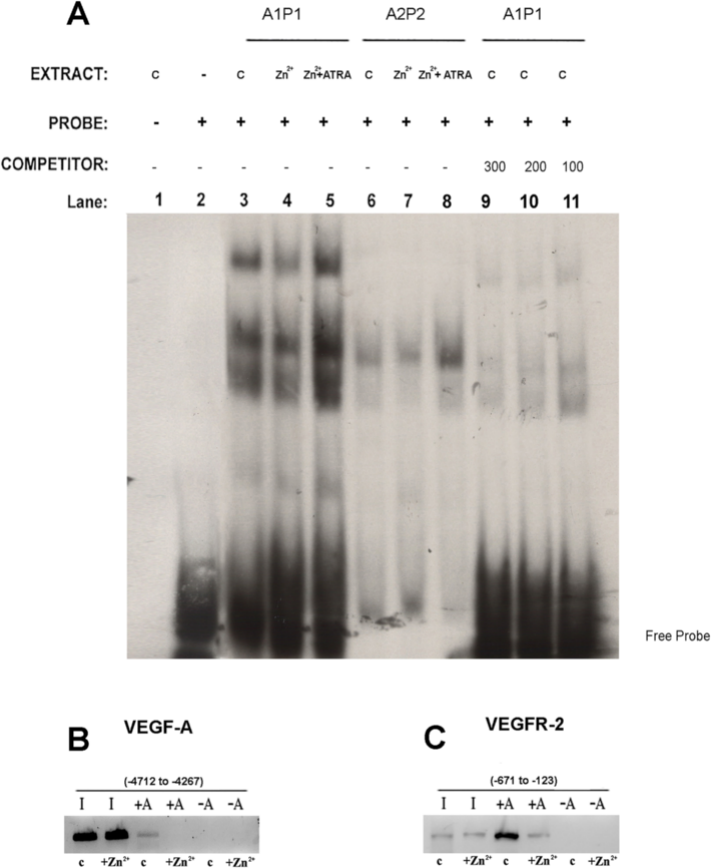
**a** Gel shift assay of cellular extracts incubated in vitro with oligonucleotides corresponding to a region of the VEGFR-2 gene promoter containing two putative HHEX binding sites. In some experiments, labeled oligonucleotide A1P1 containing two HHEX binding sites or labeled oligonucleotide A2P2 containing one HHEX binding site has been incubated with cellular extracts derived from PR9 cells grown either without additives (**c**) or for 24 h in the presence of Zn^2+^ or Zn^2+^ + ATRA. The specificity of the bands was tested adding a 100-, 200-, or 300-fold excess of unlabeled free oligonucleotide probe. **b**, **c** HHEX binding to the VEGF (**b**) and VEGFR-2 (**c**) promoters as shown by ChIP experiments. Nuclear extracts derived from PR9 cells grown for 24 h either in the absence (**b**) or in the presence of ZnSO_4_ (Zn^2+^); cells were crosslinked in vivo; after cell lysis, chromatin fragments were immunoprecipitated with anti-HHEX antibody; and after DNA purification, DNA regions containing either VEGF or VEGFR-2 were amplified. In the figure, the VEGF or VEGFR-2 PCR signal without immunoprecipitation is indicated as −A (IgG control) and after immunoprecipitation as +A (HHEX antibody)

We then evaluated whether the reduced HHEX expression induced by PML-RARα determines a reduced capacity of this transcription factor to bind at the level of target gene promoters. Thus, gel shift experiments were carried out using as target two putative HHEX binding sequences, containing core 5′-ATTA-3′ motifs, observed at the level of the VEGFR-2 promoter, which are located approximately 1.1 Kb upstream of the first exon. Cell extracts derived from PR9 cells incubated with labeled oligonucleotides corresponding to these sequences were shown to be capable to form two specific bands that were competed by 200 fold excess of cold oligonucleotides (Fig. 5a). The intensity of these two bands decreased in gel shift assays performed using cell extracts derived from Zn^2+^-treated cells, compared to untreated cells; a normal binding activity was observed in cell extracts derived from cells incubated with Zn^2+^ and ATRA (Fig. 5a).

To provide further evidence that HHEX was able to bind to VEGF and VEGFR-2 promoter and that its DNA-binding capacity was downmodulated by PML-RARαwe carried out chromatin immunoprecipitation experiments (CHIP). A genomic region of about 500–600 bp containing the HHEX binding sequence in the VEGF or the VEGFR-2 promoter was amplified in the immunoprecipitates by PCR using specific primers flanking the HHEX binding motif. In the presence of a control IgG (-Ab), no PCR product was produced. In the presence of anti-HHEX antibody, the CHIP experiments showed that under control, unstimulated conditions, the VEGF and VEGFR-2 promoters are occupied by HHEX; this binding markedly declines in PR9 cells grown for 12 h in the presence of 100 μM ZnSO_4_ (Fig. 5b, c)

### HHEX downmodulation is required for the growth-promoting, pro-angiogenetic, and differentiation blocking effects mediated by PML-RARα

In a last set of experiments, we have evaluated the possible functional consequences of PML-RARα-induced HHEX downmodulation in the context of the leukemic cell phenotype. To this end, we have used a specific small interfering RNA (siRNA) to downmodulate HHEX expression into PR9 cells; as a control, we have used a scrambled control siRNA.

**Fig. 6:**
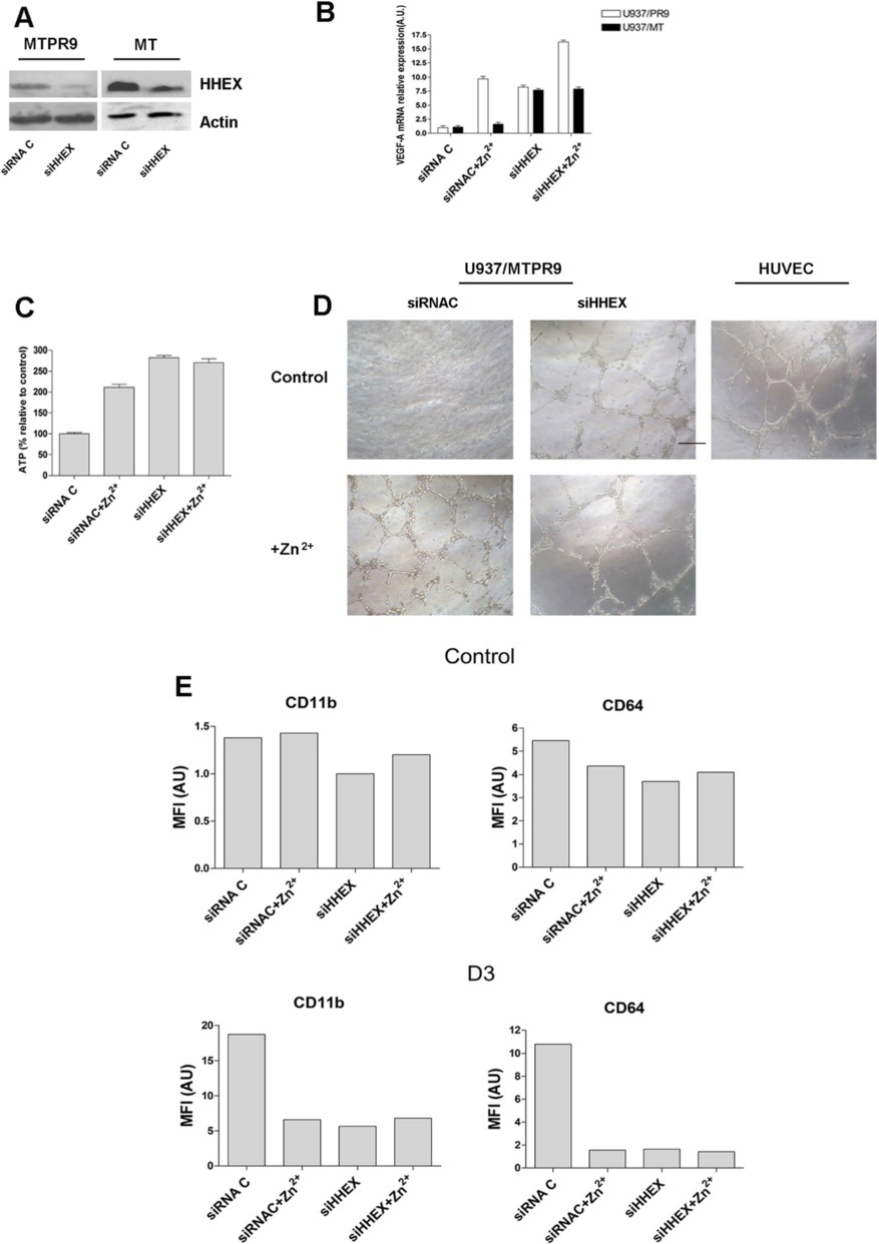
**a**, **b**, **c** Effect of HHEX silencing on cell proliferation and VEGF-A expression by PR9 cells. MT or PR9 cells have been treated either with a control siRNA or with a siHHEX RNA and then evaluated for HHEX expression by WB (**a**), VEGF-A mRNA expression by real-time PCR (**b**), and cell proliferation (**c**). **d** Effect of HHEX silencing on the capacity of PR9 cells to release pro-angiogenic growth factors. PR9 cells have been treated with a control siRNA or with siHHEX RNA and then grown for 48 h without (control) or with ZnSO_4_ (+Zn^2+^); cell supernatants were collected and assayed for their capacity to promote endothelial tube formation using an endothelial tube formation assay using HUVEC cells plated in Matrigel. As a positive control, HUVEC cells were plated in Matrigel in EGM2 complete medium containing exogenous angiogenetic growth factors. **e** Effect of HHEX silencing in the VitD3-induced cell differentiation of PR9 cells. PR9 cells were treated either with control siRNA or with siHHEX RNA and then grown for 3 days either in the absence (**c**) or in the presence of 100 μM ZnSO_4_ (Zn^2+^), either in the absence (control) or in the presence of 1α25OH-VitD3 (D3) for 72 h, harvested and then analyzed for the expression of CD11b and CD64 membrane markers by flow cytometry. The results of one representative experiment are reported in the figure

In a first experiment, we have evaluated the effect of HHEX silencing on VEGF-A production and cell growth of PR9 cells. First, we observed that HHEX silencing elicited a pronounced decrease of HHEX protein both in MT and PR9 cells, as shown by Western blotting experiments (Fig. 6a). Then, we showed that HHEX silencing was able to induce a significant increase of VEGF-A expression, comparable to that elicited by Zn^2+^ addition; the concomitant addition of Zn^2+^ and HHEX silencing elicited an additive effect on VEGF-A expression, achieving a level of expression higher than that observed in cells undergoing either type of single treatment (Fig. 6b). In parallel, we have evaluated the effects on PR9 cell growth and we observed that HHEX silencing mimicked the stimulatory effect elicited by PML-RARα on the growth of U-937 cells (Fig. 6c). Combined HHEX silencing and PML-RARα induction did not further stimulate PR9 cell growth (Fig. 6c).

In a second set of experiments, we evaluated the effect of HHEX silencing on the pro-angiogenetic capacity of PR9 cells. To this purpose, we evaluated the capacity of cell culture supernatants of PR9 cells untreated or treated for 48 h with Zn^2+^ or of HHEX-silenced cells to promote tube formation of HUVEC cells. These experiments showed that supernatants of Zn^2+^-treated or HHEX-silenced PR9 cells, but not of control PR9 cells, were able to promote HUVEC tube formation (Fig. 6d).

In a third set of experiments, we evaluated the effect of HHEX silencing on monocytic differentiation of U-937 cells induced by 1α25OH-VitD3. Previous studies showed that PML-RARα expression in U-937 cells elicited a marked inhibition of their capacity to differentiate in response to 1α25OH-VitD3 [18]. In these experiments, monocytic differentiation was monitored through the study of two membrane monocytic markers, CD11b and CD64, whose expression is strongly induced by 1α25OH-VitD3. We observed that HHEX silencing elicited an inhibition of 1α25OH-VitD3-induced monocytic differentiation of U-937 cells, comparable to that elicited by induction of PML-RARα expression, and silencing of HHEX did not result in a further inhibition of CD11b and CD64 expression, compared to the values observed with single treatment (Fig. 6e). Furthermore, morphologic analysis showed that, while the large majority of PR9 cells grown in the presence of 1α25OH-VitD3 acquire the morphology of mature monocytic elements, PR9 cells treated with Zn^2+^ or with a HHEX siRNA and then grown with 1α25OH-VitD3 remained in large part blocked at a promonocytic stage of cell differentiation (data not shown).

### HHEX overexpression inhibits the pro-angiogenetic and differentiation blocking effects of PML-RARα

**Fig. 7:**
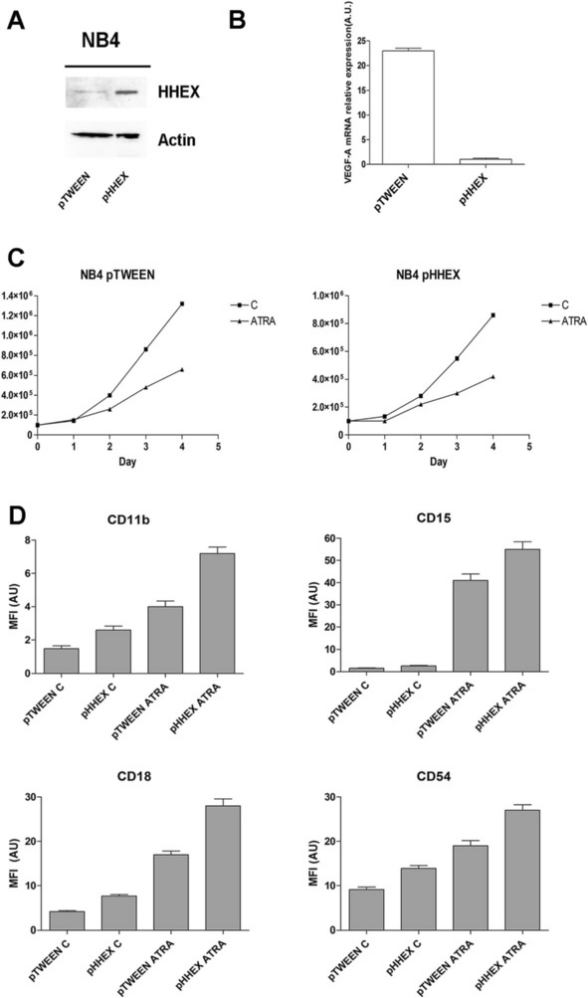
Effect of HHEX overexpression on the capacity of NB4 cells to express VEGF-A and to differentiate in response to ATRA. NB4 cells have been transfected with a control empty vector (pTWEEN) or with this vector containing the human HHEX gene (pHHEX) and then **a** analyzed for HHEX expression by Western blotting (*top panel*, *left*) and for VEGF-A RNA expression by realtime-PCR (*top panel*, *right*); **b** pTWEEN NB4 cells and pHHEX NB4 cells have been grown in the absence or in the presence of 1 μM ATRA and evaluated at different times for **c** the number of viable cells (*middle panels*) and for **d** the expression of CD11b, CD15, CD18, and CD54 membrane antigens (*low panels*). The results of the three experiments ± SEM are reported in the figure. For NB4 cells grown in the absence of ATRA, the expression level of CD18 and CD54 antigens between pTWEEN and pHHEX cells was significant (*p* ≤ 0.05). For NB4 cells grown in the presence of ATRA, the difference of CD15 (*p* ≤ 0.05) and CD11b, CD18, and CD54 (*p* ≤ 0.01) between pTWEEN and pHHEX cells was significant

**Fig. 8:**
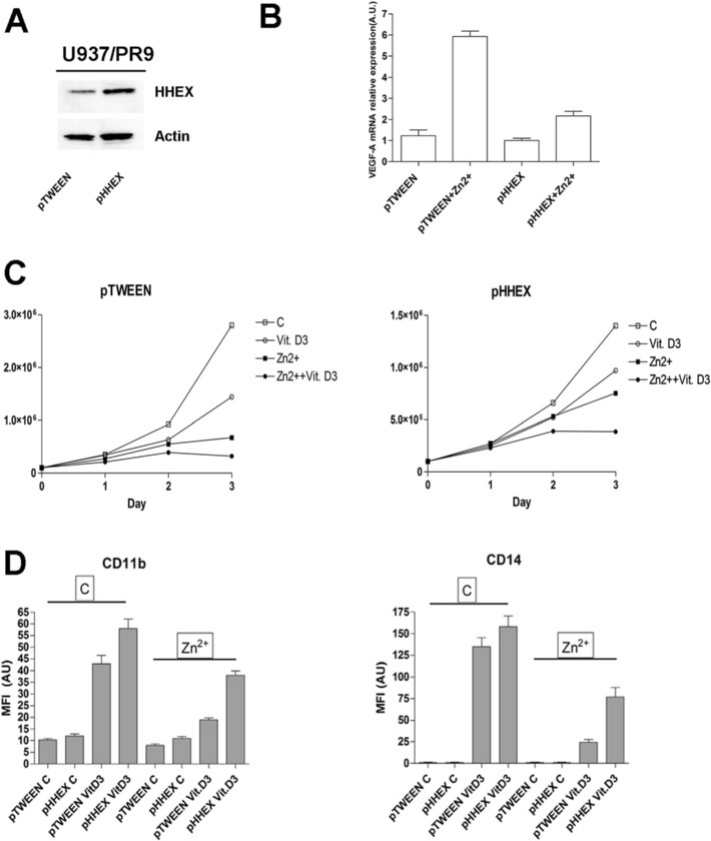
Effect of HHEX overexpression on the capacity of PR9 cells to express VEGF-A and to differentiate in response to VitD3. PR9 cells have been transfected with a control empty vector (pTWEEN) or with this vector containing the human HHEX gene (pHHEX) and then **a** analyzed for HHEX expression by Western blotting (*top panel*, *left*) and for VEGF-A RNA expression by realtime-PCR (*top panel*, *right*); **b** pTWEEN PR9 cells and pHHEX PR9 cells have been treated or not with Zn^2+^ for 24 h and then grown in the absence or in the presence of 1α25OH-VitD3 and evaluated at different times for the number of viable cells (*middle panels*) and at day 3 of culture for the expression of CD11 and CD14 membrane antigens (*low panels*). The results of the three experiments ± SEM are reported in the figure. For PR9 cells treated with Zn^2+^+VitD3, the difference between pTWEEN PR9 and pHHEX PR9 cells for both CD11b and CD14 expression was highly significant (*p* ≤ 0.01)

In a fourth set of experiments, we evaluated the effect of HHEX overexpression on the pro-angiogenetic capacity of both NB4 and PR9 cells. To this purpose, we evaluated the effect of HHEX overexpression on VEGF-A mRNA expression and we observed that (a) in NB4 cells, HHEX overexpression (Fig. 7a) elicited a marked inhibitory effect on VEGF-A mRNA levels, compared to the levels observed in the same cells transfected with a control empty vector (Fig. 7b); (b) in PR9 cells transfected with the HHEX expressing vector (Fig. 8a), Zn^2+^ addition elicited a stimulation of VEGF-A mRNA expression markedly lower than in the same cells transfected with the empty vector (Fig. 8b). HHEX overexpression moderately reduced the cell growth of both NB4 and PR9 cells, either when grown in the absence or in the presence of cell differentiation inducers, compared to the corresponding control cells transfected with the empty vector (Figs. 7c and 8c).

In a fifth set of experiments, we evaluated the effect of HHEX overexpression on the differentiation capacity of both NB4 and PR9 cells. In NB4 cells, HHEX overexpression elicited a moderate, but significant, improvement of both spontaneous and ATRA-induced granulocytic differentiation, as assessed on the basis of the evaluation of the expression of CD11b, CD18, CD15, and CD54 membrane antigens (Fig. 7d).

In PR9 cells transfected with the empty vector (pTWEEN) PML-RARα expression triggered by Zn^2+^, a marked inhibition of VitD3-induced monocytic differentiation, as assessed through the analysis of CD11b and CD14 membrane antigens, was observed (Fig. 8d). In PR9 cells overexpressing HHEX, induction of PML-RARα only moderately inhibited VitD3-induced monocytic differentiation (Fig. 8d).

## Discussion

APL is characterized by the PML/RARα fusion gene underlying the *t*(15;17) translocation and resulting in the formation of the PML-RARα oncoprotein. The molecular mechanism of leukemic transformation induced by PML-RARα is largely related to the capacity of this fusion oncoprotein to act as a potent transcriptional repressor of RARA and non-RARA target genes, thus interfering with gene expression programs required for hemopoietic progenitor self-renewal and myeloid cell differentiation [19, 20].

APL blasts are blocked at the promyelocytic stage of myeloid cell differentiation and two unrelated agents, ATRA and arsenic trioxide, are able to induce their differentiation both in vitro and in vivo. ATRA induces granulocytic differentiation through a molecular mechanism mainly involving transcriptional reactivation of PML-RARα-silenced genes [21], while ATO acts through induction of PML/RARα degradation, the clearance of PML-RARα-bound promoters being sufficient to induce APL differentiation [22].

In line with these observations, in the present study, we provided evidence that PML-RARα inhibits HHEX expression in APL cells. This downmodulation of HHEX expression, compared to the normal physiological counterpart of APL, was constantly observed in all the 18 primary APLs investigated in the present study. Experiments carried out in U-937 cells expressing inducible PML-RARα strongly support a direct role of this fusion protein in inhibiting HHEX expression. The reduced HHEX expression seems to have two relevant functional consequences in APL cells. First, it seems to be responsible, at least in part, for the elevated expression of angiogenetic factors in APLs. This conclusion is supported by three lines of evidence: in fresh APL blasts, we observed a significant inverse correlation between HHEX and VEGF-A levels; HHEX silencing in PR9 cells mimics the stimulatory effect of PML-RARα on VEGF-A expression; HHEX overexpression in both NB4 and PR9 cells markedly inhibits PML-RARα-induced VEGF-A expression. Our observations on the inverse relationship between HHEX and VEGF-A levels in primary APL cells were corroborated through the analysis of gene expression data available for various AMLs on the TCGA platform. Interestingly, this analysis showed also that among the various AMLs, APLs display the highest VEGF-A expression and the lowest HHEX/VEGF-A ratio. These observations further support numerous other literature data showing a particularly elevated expression of angiogenetic growth factors in APLs [23–25]. The key and a direct involvement of HHEX in transcriptional regulation of multiple genes encoding components of the VEGF signaling pathway, including VEGF, VEGFR-1, and VEGFR-2, was also shown in a chronic myeloid leukemia model [26]. It is of interest to note that among the various AML subtypes, M5 AMLs are characterized by low HHEX levels and also by increased VEGF-A levels, although less elevated than in M3 AMLs. An inverse relationship between HHEX and VEGF-A was observed also in M5 AMLs. Interestingly, a previous study showed that M5 AMLs were characterized not only by decreased HHEX levels but also by a delocalization of HHEX protein that, at variance with other cell types, is largely confined to the cytoplasm [27].

The stimulation of an angiogenetic response in APL cells induced by PML-RARα seems to be relevant for disease pathogenesis. This conclusion is supported by recent studies showing that angiogenesis inhibitors [28] and HIF inhibitors [29] impair leukemia progression and prolong mice survival in APL mouse models.

Second, the reduced HHEX expression induced by PML/RARα in APL cells seems to play a relevant role in mediating the inhibitory effect of this fusion protein on cell differentiation. This conclusion is supported by experiments carried out on NB4 and PR9 cells. As previously reported, induction of PML-RARα expression in PR9 cells resulted in a marked inhibition of their capacity to differentiate into monocytes in response to 1α25OH-VitD3 [18]. Interestingly, the silencing in these cells of HHEX expression by siRNA resulted in an inhibitory effect on 1α25OH-VitD3-induced cell differentiation, highly comparable to that induced by PML-RARα. In contrast, HHEX overexpression in these cells resulted in a partial rescue of the inhibitory effect elicited by PML-RARα on monocytic differentiation.

It is of interest to note that recently Kramarzova and coworkers reported a large screening of homeobox cluster A and B expression at the level of various AML FAB subtypes and clearly showed that the lowest levels were observed in FAB M3 AMLs [30]. Particularly, HOXA1, HOXA5, HOXA6, and HOXB6 expression was low in APLs [30]. Combined to the findings of the present study, these observations strongly suggest a general repressive role of the PML/RARα fusion gene on HOX gene expression.

A low HHEX expression was recently reported also in AMLs characterized by trisomy 8, the most frequent chromosome numeric aberration observed in AMLs [31]. The HHEX gene was hypermethylated in trisomy 8 AMLs, a finding in line with the low HHEX expression observed in these AMLs [31]. In this study, 3 APLs were studied and all three displayed a hypermethylation of HHEX gene [31].

The present study contributes in part to clarify the complex mechanisms through which PML-RARα exerts its oncogenic potential. In this context, recent studies highlight a relevant role of some long non-coding RNAs (lncRNAs) in the mediation of some key pathogenetic events induced by PML-RARα, particularly at the level of the control of cell proliferation [32] and of cell differentiation [33].

The identification and characterization of biomarkers is fundamental in oncology and represents an essential tool for drug development [34]. At the moment, no data support a potential role of HHEX as a disease biomarker for APLs. However, future studies will address this important issue.

## Methods

### Human progenitor cell (HPC) purification

Cord blood (CB) was obtained after informed consent from healthy full-term placentas according to institutional guidelines. Human CD34+ cells were purified from CB by positive selection using the midi-MACS immunomagnetic separation system (Miltenyi Biotec, Bergisch Gladabach, Germany) according to the manufacturer’s instructions. The purity of CD34+ cells was assessed by flow cytometry using a monoclonal PE-conjugated anti-CD34 antibody and was routinely over 95 % (range comprised between 92–98 %). Purified human hematopoietic progenitor cells were grown in serum-free medium containing BSA (10 mg/ml), pure human transferrin (1 mg/ml), human low-density lipoproteins (40 μg/ml), insulin (10 μg/ml), sodium pyruvate (10–4 M), l-glutamine (2 × 10^−3^ M), rare inorganic elements (Sn, Ni, Va, Mo, and Mn) supplemented with iron sulfate (4 × 10^−8^ M), and nucleosides (10 μg/ml each). HPCs were induced into specific granulopoietic differentiation with IL-3 (1 unit/ml), granulocyte/monocyte CSF (0.1 ng/ml), and saturating amounts of G-CSF (500 units/ml). The differentiation stage of unilineage cultures was evaluated by MayGrunwald–Giemsa staining (Sigma-Aldrich, St. Louis, MO, USA) and cytologic analysis.

### Primary leukemic cells

AML fresh leukemic blasts were isolated from diagnostic bone marrows obtained from 18 patients with newly diagnosed APL, using Ficoll–Hypaque density gradient. The rapid diagnosis of APL was based on the detection by immunofluorescence microscopy of a microspeckled nuclear pattern that is characteristic of PML protein delocalization from nuclear bodies in APL. The diagnosis was confirmed by cytogenetic evidence of the *t*(15;17)(q22;q21) and/or by reverse transcription polymerase chain reaction (RT-PCR) detection of the PML-RARα rearrangement on BM samples. According to the Declaration of Helsinki, informed consent was obtained from all patients and healthy donors and the study was approved by the Institution Review Board (IRB) of the University of Tor Vergata of Rome, Italy.

### Cell line culture and differentiation

Fresh leukemic blasts were isolated from either bone marrow (BM) or peripheral blood (PB) by Ficoll–Hypaque density centrifugation obtained after informed consent from 18 APL patients classified as M3 by morphological criteria according to the French–American–British (FAB) classification. APL cells were grown in the above medium in the absence (control) or in the presence of ATRA (1 × 10^−6^ M; Sigma, St Louis, MO, USA).

The AML cell lines NB4, U937-MT, and U937-PR9 cells (described subclones of the U937 promonocytic cell line [17]) were grown in RPMI 1640 medium supplemented with 10 % fetal calf serum in 5 % CO_2_, 95 % humidified air at 37 °C. In some experiments, NB4 cells were treated for 3 days with 1 μM ATRA (Sigma). U937-PR9 and U937-MT cells were used for induction of PML-RARα expression or as control, respectively. Cells were cultured either with or without varying concentrations of ZnSO_4_ (Sigma), ATRA (Sigma), or 50 ng/ml 1α250H-Vitamin D3 (Roche, Basel, Switzerland) for the indicated time points. After different days of in vitro incubation, cells were harvested and assayed for cell vitality (by the trypan blue exclusion test) and cell differentiation. Cell differentiation was assessed by cell morphology and changes in cell surface antigen expression.

Packaging cell line, 293FT, was maintained in DMEM (Life Technologies Corporation, Carlsbad, CA, USA) supplemented with 10 % (*v*/*v*) heat-inactivated FBS, 2 mM l-glutamine, 100 U/ml penicillin, and 100 ug/ml streptomycin (Invitrogen).

### FACS analysis

Analysis of cell surface antigens was performed by flow cytometry using a FACScan Flow cytometer (Becton Dickinson, Bedford, MA, USA). The cells were stained with a PE-conjugated anti-mouse, CD11b, CD14, CD15, CD18, CD54, and CD64 antibody (Pharmingen, San Diego, CA, USA) as previously reported. Briefly, the cells were incubated for 30 min at 4 °C with an appropriate dilution of antibody; and after three washings in PBS, the cells were fixed in PBS formaldehyde (4 %) and analyzed by flow cytometry. The results were expressed in terms of the percentage of positive cells and of the mean fluorescence intensity (MFI).

### Western blot analysis

To prepare total extracts, the cells were washed twice with cold phosphate-buffered saline and lysed on ice for 30 min with 1 % Nonidet P40 lysis buffer (20 mM Tris–HCl pH 8.0, 137 mM NaCl, 10 % glycerol, 2 mM EDTA) in the presence of 1 mM phenymethylsulfonyl fluoride, 1 mM dithiothreitol, 1 mM sodium orthovanadate, 2 μg/ml leupeptin, and 2 μg/ml aprotinin. Cell debris was removed by centrifugation at 10,000 rpm for 10 min at 4 °C, and protein concentration of supernatants was determined by the Bio-Rad protein assay (Richmond, CA, USA). Aliquots of cell extracts containing 30–50 μg of total protein were resolved by 7.5–10 % SDS-PAGE under reducing and denaturing conditions and transferred to nitrocellulose filter. The blots were blocked using 5 % non-fat dry milk in TBST (10 mM Tris–HCl pH 8.0, 150 mM NaCl, 0.1 % Tween 20) for 1 h at room temperature, followed by incubation with primary antibodies. After washing with TBST, the filters were incubated with the appropriate horseradish-peroxidase-conjugated secondary antibodies (Bio-Rad) for 1 h at room temperature. Immunoreactivity was revealed using an ECL detection kit (Pierce, USA). In Western blot experiments, the following antibodies were used: HHEX (Epitomics); VEGFR-2 and RARα (Santa Cruz Biotechnology); actin (Oncogene Research Products, Cambridge, MA),

### Cell transfection and proliferation

Transient transfections of U937-PR9 cells with siRNA were carried out using Lipofectamine 2000 (Invitrogen, Carlsbad, CA, USA). Chemically synthesized siRNAs (Silencer Select Pre-designed and Validated siRNA) to HHEX, and scrambled siRNAs were purchased from Ambion and transfected at 10 nM final concentration. After 24 h, the cells were treated with no additives (control) or 50 ng/ml VitD3 or 1 μM Zn^2+^ or both drugs at the above doses. After 72 h, the expression of HHEX was assayed by Western blot. Cell growth was analyzed using Cell Titer-GloMax assay (Promega, Fitchburg, WI, USA).

### Plasmid constructs and lentivirus infection

HHEX complementary DNA (cDNA) (NM_002729) was synthesized and cloned in Xhol-EcoRV restriction sites under CMV promoter into a variant third-generation lentiviral vector, pRRL-CMV-PGK-GFP-WPRE, called Tween [35], to simultaneously transduce both the reporter GFP and the gene HHEX. Lentiviral particles were produced in 293FT packaging cell line and infection performed as previously described [35]. After infection, transduced cells were selected with green fluorescent protein (GFP) fluorescence evaluated by FACS analysis.

### RNA extraction and analysis

Total RNAs were extracted by the guanidinium isothiocyanate-CsCl method and reverse transcribed using random primers-RT kit (Invitrogen), according to the manufacturer’s procedure. The RT-PCR was normalized for S26 ribosomal protein. The sequences of the oligonucleotide primers used for RT-PCR were as follows: S26 5′-GCCTCCAAGATGACAAAG-3′ and 5′-CCAGAGAATAGCCTGTCT-3-; VEGFR-2 5-GTGACCAACATGGAGTCGTG-3′ and 5′-CCAGAGATTCCATGCCACTT-3′; HHEX 5′-TTCTCCAACGACCAGACCATCG-3′ and 5′-TTTTATCGCCCTCAATGTCCAC-3′; VEGF-A GGCTCTAGATCGGGCCTC and GGCTCTAGAGCGCAGAGT.

Samples were electrophoresed in 1.5 % agarose gel, transferred onto Hybond-N (Amersham Pharmacia Biotech, Uppsala, Sweden) filter and hybridized with an internal oligomer probe.

Quantitative real-time (qRT)-PCR analysis was performed by TaqMan technology, using the ABI PRISM 7700 DNA Sequence Detection System (Applied Biosystems, Foster City, CA, USA). Commercial ready-to-use primers/probe mixes were used (Assays on Demand Products, Applied Biosystems) for GAPDH, HHEX, VEGF-A and VEGFR-2, Tie 2, angiopoietin-1, and FGFR-1.

### Chromatin immunoprecipitation (ChIP)

Chromatin immunoprecipitation (ChIP) assay was performed according to Upstate Biotechnology protocol (Lake Placid, NY, USA). Briefly, 5 × 10^6^ cells were crosslinked in vivo, lysed, and immediately sonicated. Chromatin fragments were immunoprecipitated with 4 μg of anti-HHEX (Epitomics) or anti-RARα (SantaCruz)  antibody; and after treatment with proteinase K for 2 h at 68 °C, the DNA was purified by phenol/chloroform extraction and amplified by Hot-start PCR (30 cycles of 94 °C for 1 min, 60 °C for 1 min, and 72 °C for 1 min) using the following primers:

VEGFR-2 (FWD) 5′CCTTCTTGGGGCTAGGCAGGTCACTTCA3′ (−671 to −644), and VEGFR-2 (REV), 5′GATCTCCAGCTCCCCAAGCCCATTTA3′ (−148 to −123).

VEGF (FWD) 5′AAAGACCCAACTCAAGTATCATCTSSAGT3′, and VEGF (REV) 5′CACTCACTGTGTGTGGCCTTAGGTTATTCAAC3′.

HHEX (FWD) 5′GGTTCAACAGGTTTGTGCAGT3′ and HHEX (REV) 5′CCGGCTATCAGAAGTCGAGTG3′.

### EMSA

The double-stranded HHEX binding sites (400 ng) were labeled with [α32]P dATP using Klenow enzyme. The following sequences in the VEGFR-2 receptor promoter containing a HHEX consensus site (5-ATTA-3′) were used:

A1P1: 5′-GCCATATACATTCATTATATTTCAGCATTAAAATATTTC-3′ and 5′-TATATGTAAGTAATATAAAGTCGTAATTTTATAAAG-3′.

A2P2: 5′-TTCGGGGACCGGCAAGCGATTAAATCTTGGAGTTGCT-3′ and 5-AAGCCCCTGGCCGTTCGCTAATTTAGAACCTCAACGA-3′.

The binding-reaction mixture (20-μl final volume) contained labeled oligonucleotide probes (10,000 cpm) in binding buffer (75 mM KCI, 20 mM Tris–HCl (pH 7.5), 1 mM dithiothreitol) containing 5 μg of bovine serum albumin per milliliter, 14 % (vol/vol) glycerol, and 3 μg of poly(dI-dC). Total cell lysates (10 μg) were added, and the reaction mixture was incubated for 20 min at room temperature. Samples were electrophoresed in a 5 % poly-acrylamide gel in 0.5× Tris-borate-EDTA (TBE) buffer for 2 h at 200 V at 18 °C. The gels were then dried and subjected to autoradiography. Competition studies were performed by adding unlabeled double-stranded oligonucleotides at a 100-, 200-, and 300-fold molar excess over the labeled probe.

### Analysis of TCGA data

Gene expression analysis data from AML samples were collected as previously described (The Cancer Genome Atlas Research Network 2008). Processed data sets were obtained directly from the public access data portal (http://cancergenome.nih.gov/dataportal/data/about); 176 AML samples stratified by leukemia French American British morphology classification (FAB) and a total of 20,319 genes with expression values in the RPKM format were included. The expression levels of HHEX and VEGF-A of the 179 samples from 6 FAB categories were plotted. Data sets were cross-referenced using tumor-specific identification numbers.

### Matrigel in vitro HUVEC tube formation assay

PR9 cells treated in different conditions, as reported in the corresponding section “Results”, have been grown for 48 h, and the conditioned media were collected, centrifuged, transferred to fresh tubes, and stored at −20 °C. Growth factor-reduced Matrigel (100 μl), after being thawed on ice, was plated in a 96-well cell culture plate. The chamber was then incubated at 37 °C for 30 min to allow the Matrigel to polymerize. HUVEC maintained in complete EGM2 medium and starved for 4 h were trypsinized and seeded (2 × 10^4^ cells/well) in each well with 100 μl of conditioned medium. EGM2 complete medium, supplemented with endothelial growth factors, was used as a positive control, while EM2 medium without growth factors was used as a negative control. The multiwell plates were incubated for 24 h and then tube formation was analyzed through inspection using and inverted microscope equipped with a digital camera.

### Statistical analysis

Data were analyzed using parametric statistics with one-way analysis of variance (ANOVA). Post hoc tests included the Student’s *t* test and the Tukey multiple comparison tests as appropriate using Prism (GraphPad, San Diego, CA, USA). Data are presented as mean value ± SEM from three independent experiments. Significance was set at *p* < 0.05.

## Conclusions

HHEX downmodulation induced by PML-RARα is a key event during APL pathogenesis and is physiopathologically relevant to mediate the inhibitory effect on cell differentiation and the pro-angiogenetic effects elicited by this oncogenic fusion protein.

## Additional file

Additional file 1: Figures S1A, S1B, and S1C. Figure S1A Analysis of HHEX (*top panel*) and VEGF-A (*middle panel*) reported in 176 primary AMLs on the TCGA platform. The HHEX/VEGF-A ratio is reported in the bottom panel. Figure S1B Correlation between HHEX and VEGF-A levels observed in 18 primary APLs in the present study (*p* = 0.0484) and in 16 primary APLs in the TCGA data (*p* = 0.0284). Figure S1C Correlation between HHEX and VEGF-A levels observed in 18 primary APLs (*p* = 00484) and in 20 primary M5 AMLs in the TCGA data (*p* = 0.0174). (ZIP 689 kb)
