# Supplementary figures and images for: PML-RAR alpha induces the downmodulation of HHEX: a key event responsible for the induction of an angiogenetic response
Source: J Hematol Oncol. 2016 Apr 7;9:33. doi: 10.1186/s13045-016-0262-5 (PMC4823896; doi:10.1186/s13045-016-0262-5)

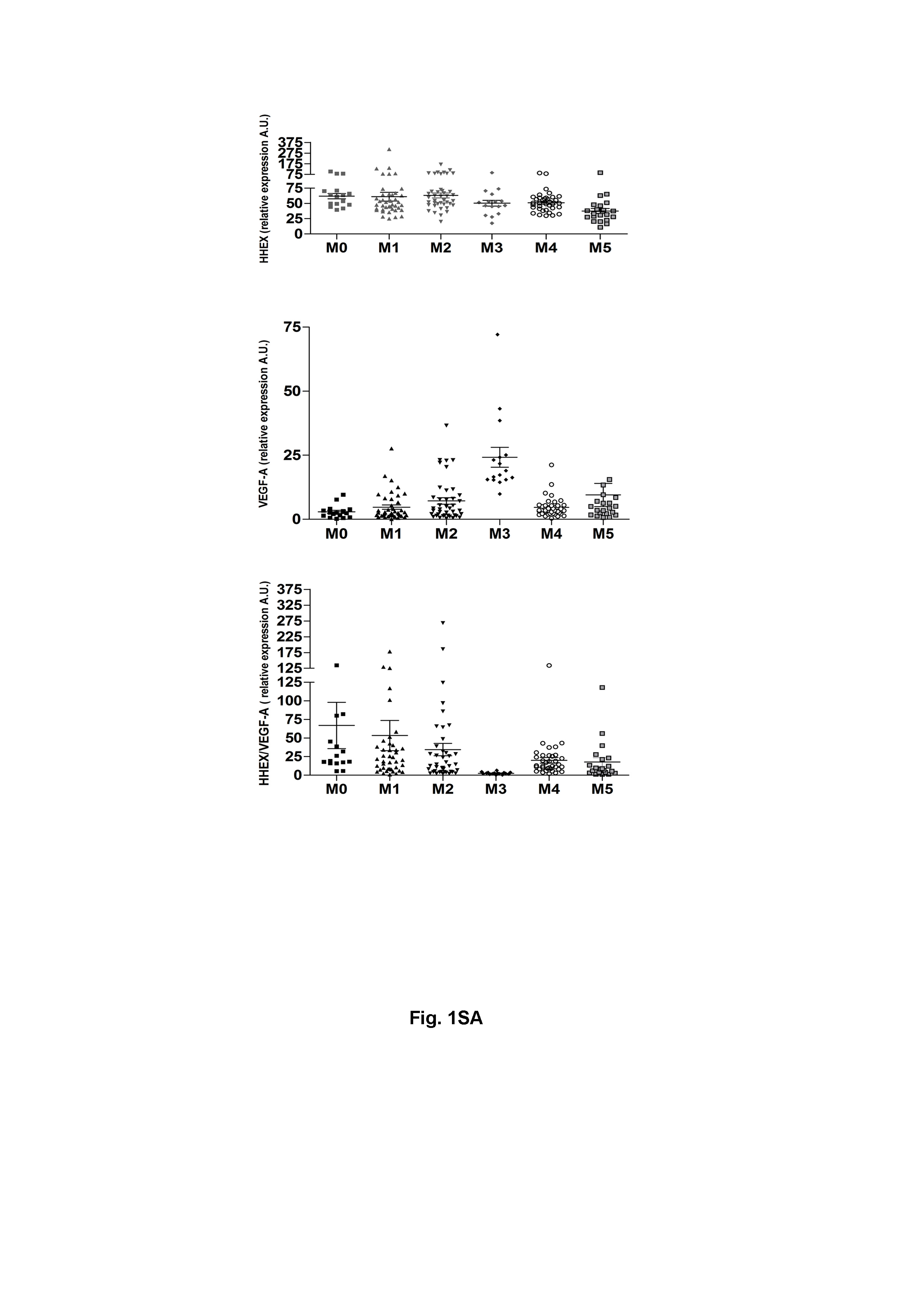

Supplement: Additional file 1: Figures S1A, S1B, and S1C. — Figure S1A Analysis of HHEX (top panel) and VEGF-A (middle panel) reported in 176 primary AMLs on the TCGA platform. The HHEX/VEGF-A ratio is reported in the bottom panel. Figure S1B Correlation between HHEX and VEGF-A levels observed in 18 primary APLs in the present study (p = 0.0484) and in 16 primary APLs in the TCGA data (p = 0.0284). Figure S1C Correlation between HHEX and VEGF-A levels observed in 18 primary APLs (p = 00484) and in 20 primary M5 AMLs in the TCGA data (p = 0.0174). (ZIP 689 kb) [file 13045_2016_262_MOESM1_ESM.zip › Figure S1A-C/fig 1SA.jpg]

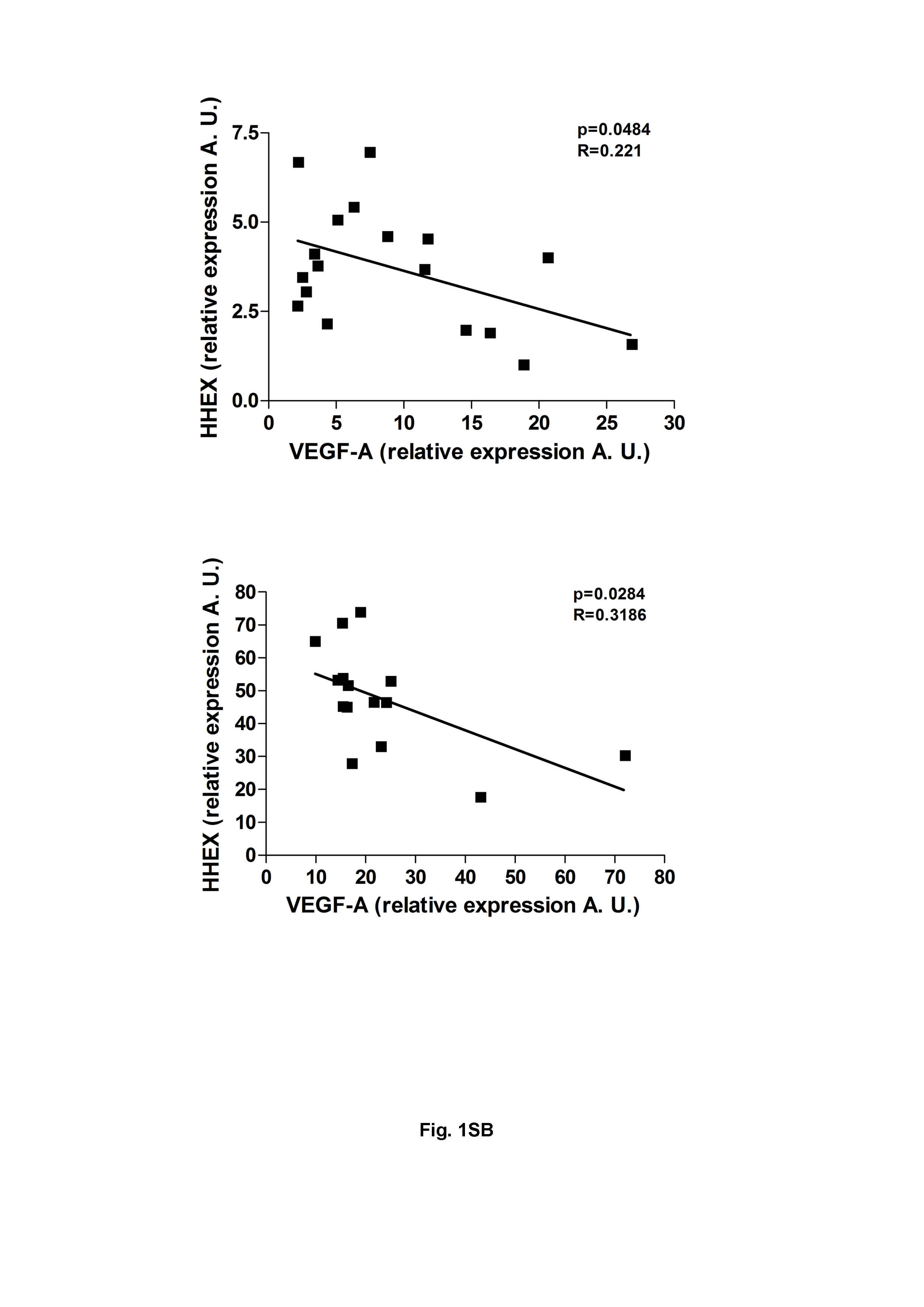

Supplement: Additional file 1: Figures S1A, S1B, and S1C. — Figure S1A Analysis of HHEX (top panel) and VEGF-A (middle panel) reported in 176 primary AMLs on the TCGA platform. The HHEX/VEGF-A ratio is reported in the bottom panel. Figure S1B Correlation between HHEX and VEGF-A levels observed in 18 primary APLs in the present study (p = 0.0484) and in 16 primary APLs in the TCGA data (p = 0.0284). Figure S1C Correlation between HHEX and VEGF-A levels observed in 18 primary APLs (p = 00484) and in 20 primary M5 AMLs in the TCGA data (p = 0.0174). (ZIP 689 kb) [file 13045_2016_262_MOESM1_ESM.zip › Figure S1A-C/fig 1SB.jpg]

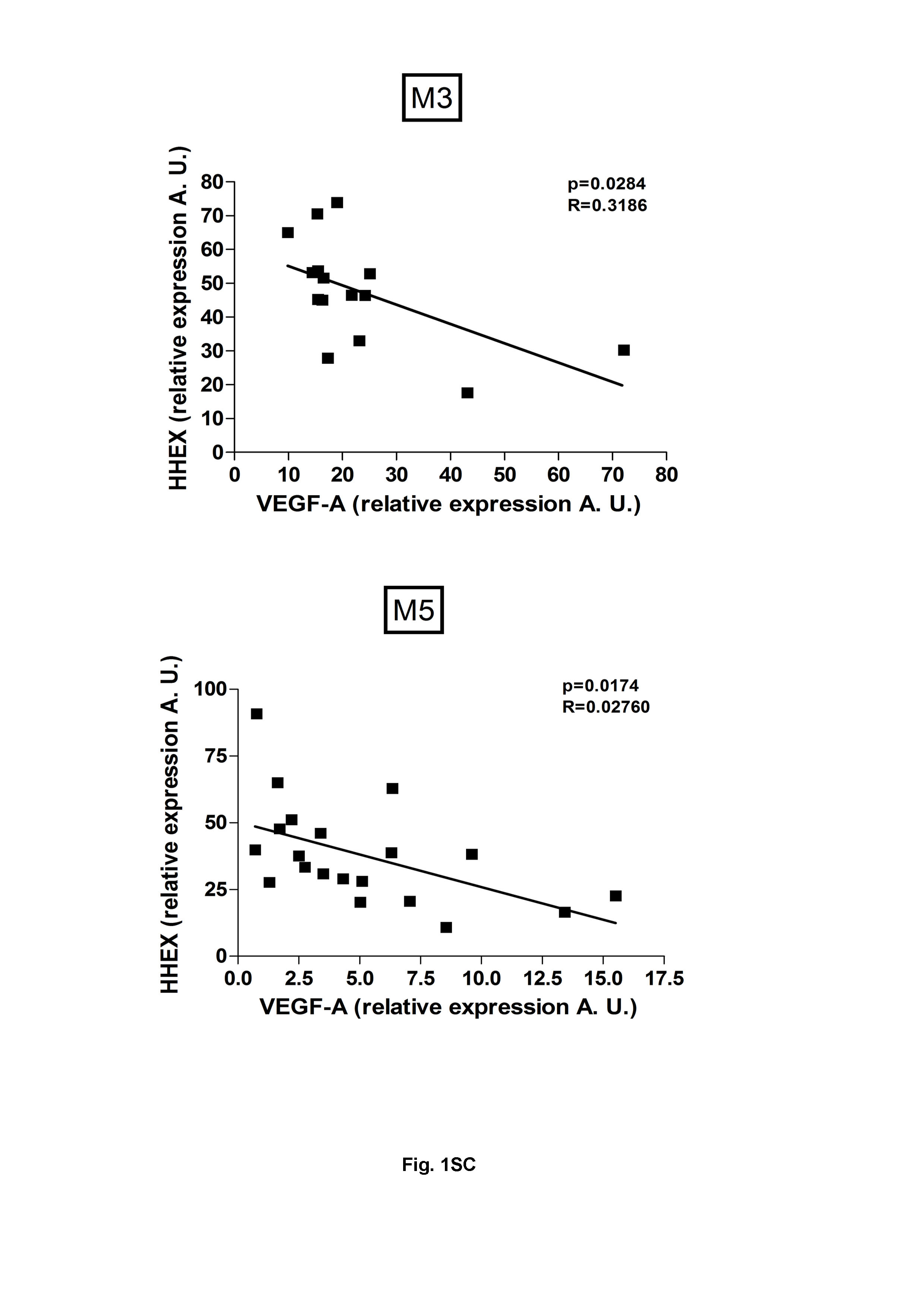

Supplement: Additional file 1: Figures S1A, S1B, and S1C. — Figure S1A Analysis of HHEX (top panel) and VEGF-A (middle panel) reported in 176 primary AMLs on the TCGA platform. The HHEX/VEGF-A ratio is reported in the bottom panel. Figure S1B Correlation between HHEX and VEGF-A levels observed in 18 primary APLs in the present study (p = 0.0484) and in 16 primary APLs in the TCGA data (p = 0.0284). Figure S1C Correlation between HHEX and VEGF-A levels observed in 18 primary APLs (p = 00484) and in 20 primary M5 AMLs in the TCGA data (p = 0.0174). (ZIP 689 kb) [file 13045_2016_262_MOESM1_ESM.zip › Figure S1A-C/fig 1SC.jpg]
